# Albumin Acts as a Lubricant on the Surface of Hydrogel and Silicone Hydrogel Contact Lenses

**DOI:** 10.3390/polym13132051

**Published:** 2021-06-23

**Authors:** Chen-Ying Su, Lung-Kun Yeh, Tzu-Wei Fan, Chi-Chun Lai, Hsu-Wei Fang

**Affiliations:** 1 Department of Chemical Engineering and Biotechnology, National Taipei University of Technology, 1, Sec. 3, Zhongxiao E. Rd., Taipei 10608, Taiwan; chenying.su@ntut.edu.tw (C.-Y.S.); sdfwer119@gmail.com (T.-W.F.); 2 Department of Ophthalmology, Chang Gung Memorial Hospital, Linkou, No. 5, Fuxing St., Taoyuan 333, Taiwan; yehlungkun@gmail.com (L.-K.Y.); chichun.lai@gmail.com (C.-C.L.); 3 College of Medicine, Chang Gung University, No. 259, Wenhua 1st Rd., Taoyuan 333, Taiwan; 4 Institute of Biomedical Engineering and Nanomedicine, National Health Research Institutes, No. 35, Keyan Road, Zhunan Town, Miaoli County 35053, Taiwan

**Keywords:** lubricant, tribology, albumin deposition, contact lens, surface roughness

## Abstract

Feeling comfortable is the greatest concern for contact lens wearers, and it has been suggested that in vivo comfort could be corresponded to the in vitro friction coefficient of contact lenses. How tear albumin could affect the friction coefficient of silicone hydrogel and hydrogel contact lenses was analyzed by sliding a lens against a quartz glass in normal and extremely high concentration of albumin solution. Albumin deposition testing and surface roughness analysis were also conducted. The results showed that the friction coefficient of tested contact lenses did not correspond to both the albumin deposition amount and surface roughness, but we proposed a model of how albumin might act as a lubricant on the surface of some hydrogel and silicone hydrogel contact lenses. In conclusion, albumin provided lubrication for silicone hydrogel contact lenses regardless of albumin concentrations, while albumin only acted as a lubricant for hydrogel contact under normal concentration.

## 1. Introduction

Tear film is composed of lipids, proteins, electrolytes, mucins, and water [1]. Once a contact lens is put into the eye, tear proteins and lipids will be instantly deposited on the surface of the lens and will easily accumulate after wearing contact lenses for a long day. Although tear proteins can protect eyes from being infected, protein accumulation caused by incomplete cleaning often can trigger immune reactions and lead to discomfort, red eyes, or contact lens-induced papillary conjunctivitis [2,3]. Many researches about protein deposition on contact lenses have been focused on lysozyme, which is the most plentiful tear protein and the main protein that gets deposited on contact lenses [4]. Lysozyme is not the only tear protein, and other tear proteins such as albumin, a natural polymer, also play a critical role in the interaction with contact lens materials [5]. The tear albumin concentration is much lower (0.02–1.1 mg/mL) than lysozyme (1.9 mg/mL) [4,6]. However, the concentration of albumin increases after wearing contact lenses or orthokeratology lenses [6,7,8]. It also has been shown that the levels of albumin rose significantly in patients with infections or dry eye, and albumin concentration could be increased to 8.3 mg/mL on average, which was 415 times higher than 0.02 mg/mL [7,9]. Therefore, the change of albumin concentration may be a critical clinical index, making albumin a protein of interest when studying protein deposition on contact lenses.

Tear protein deposition can trigger immune reactions of contact lens wearers, and it also can result in discomfort. Since the in vitro coefficient of friction (COF) of contact lenses has been shown to correspond to degree of comfort in vivo [10,11], some researchers have been focused on investigating the relationship between tear protein deposition and the COF of contact lenses. Ngai et al. showed that COFs were lower when contact lenses were exposed to a mixture of lysozyme and albumin prior to friction testing and suggested that proteins might contribute to lubricating lenses at the early stage of protein deposition [12]. Sterner et al. demonstrated that the COF of most contact lenses increased after contact lenses were repeatedly immersed in tear-like fluid and exposed to air, which may imply that the degree of comfort from wearing contact lenses changes during the course of the day [13]. Although numerous studies have focused on the relationship between protein deposition on the contact lenses and the clinical degree of comfort, there is still no direct evidence indicating a correlation [10]. We have previously shown that the COFs of some hydrogel contact lenses such as Polymacon or Hefilcon-A increased in higher concentrations of lysozyme, and a higher COF corresponded to changes in the lysozyme secondary structure rather than lysozyme deposition amounts [14,15,16].

In order to understand whether albumin deposition on contact lenses corresponded to COFs, we investigated the COF of two hydrogel (Etafilcon-A and Polymacon) and two silicone hydrogel contact lenses (Somofilcon-A and Senofilcon-A) in solutions with different albumin concentrations by using an in vitro friction testing system we established previously [15]. In addition, contact lenses were immersed in albumin before friction testing to understand the tribological properties of contact lenses if protein depositions were not removed completely. Surface roughness was also tested to provide a qualitative evaluation of the four contact lens types under investigation.

## 2. Materials and Methods

### 2.1. Contact Lenses and Reagents

The two hydrogel contact lenses used here were Etafilcon-A contact lenses (1 Day Acuvue Moist, Johnson & Johnson, New Brunswick, NJ, USA), and Polymacon contact lenses (Hydron Eye Secret Aspheric Daily, Yung Sheng Optical Co., Ltd., Taichung City, Taiwan). The two silicone hydrogel contact lenses used were Somofilcon-A contact lenses (Clariti 1 Day, Cooper Vision, Victor, NY, USA), and Senofilcon-A contact lenses with HydraLux (Acuvue Oasys Brand, Johnson & Johnson, New Brunswick, NJ, USA). Bovine serum albumin powder (Sigma-Aldrich, St. Louis, MO, USA) was dissolved in phosphate-buffered saline (PBS) for a final concentration of 0.2 or 50 mg/mL. Then, 0.2 mg/mL albumin was considered as control in this study but was higher than normal tear albumin to simulate the condition after wearing contact lenses [17]. Then, 50 mg/mL of albumin was set extremely high and could not represent the albumin level of dry eye patients, but it was investigated in this study in order to observe the influence of albumin adsorption on the tribological properties of different contact lens materials.

### 2.2. Coefficient of Friction Testing System

A CETR universal micro-tribometer-2 (UMT-2, Bruker, Campbell, CA, USA) was used for measuring COF for different contact lens materials in PBS or PBS with 0.2 or 50 mg/mL albumin. The testing system has been previously described [15]. The friction testing program used in this study was as follows; force: 0.76 kPa, rotation radius: 8 mm (mm), rotation speed: 1 revolution per minute (rpm) or 50.24 mm/minute, rotation time: 900 s. The friction force was recorded every 0.03 s by the UMT-2 sensor, and the COF was obtained by dividing friction force by normal force. The COF of each cycle (1 min) was averaged. Four independent lenses were tested for each condition.

### 2.3. Albumin Deposition Analysis

Each contact lens was placed in 3 mL of 50 mg/mL albumin solution at room temperature for 15 min. Then, the lens was taken out of the albumin solution and washed three times with 1 mL of PBS for each wash. These 3 mls of PBS from the wash were combined with the initial albumin solution. The Bio-Rad DC protein assay (Bio-Rad, Hercules, CA, USA) was used for measuring the amount of albumin in the combined solution. The optical density (OD) value was obtained by an Enzyme-Linked Immuno Sorbent Assay (ELISA) reader with a wavelength of 280 nm. The total albumin amount in the solution was determined by multiplying the concentration (mg/mL) by the total volume (6 mL). In order to determine the deposition amount onto the lens, the amount in the solution was subtracted from the starting mass, which was 150 mg (3 mL × 50 mg/mL). Three independent lenses were tested for each condition.

### 2.4. Surface Roughness Measurement

Atomic force microscopy (AFM, XE-100, Park) was used for measuring the surface roughness. The tip of AFM was PointProbe ^®^ Plus from Nanosensors (Neuchatel, Switzerland). The shape of the tip was a polygon-based pyramid, the radius of the tip was smaller than 7 nm, and the tip height was 10–15 μm. The four periphery edges of each contact lens were cut in order to create a flat surface, resulting in a 4 × 4 mm square of lens for analysis. The micro-arm was used to sense and amplify the force between the sharp probe on the cantilever and the surface of the contact lens. The probe frequency was set to a range between 0 and 1000 kilohertz, the scan range was 5 × 5 μm, and the *z*-axis range was smaller than 12 μm.

### 2.5. Statistical Analysis

The 2-tailed *t*-test was assessed in order to compare differences in albumin deposition amounts between two different lens materials. A value of *p* < 0.05 was considered significant.

## 3. Results

### 3.1. Friction Coefficient of Contact Lenses in Different Concentrations of Albumin

**Figure 1:**
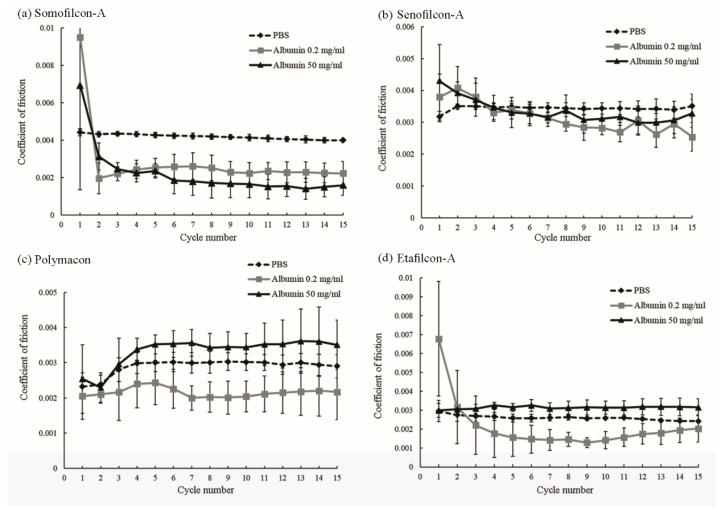
The coefficients of friction of Somofilcon-A (**a**), Senofilcon-A (**b**), Polymacon (**c**), and Etafilcon-A (**d**) lenses when sliding against glass in phosphate-buffered saline (black dashed line), 0.2 mg/mL albumin (gray line), or 50 mg/mL albumin (black line). Error bars represented standard deviation and were calculated from four experiments.

The COF of four different contact lenses in PBS or PBS with 0.2 or 50 mg/mL albumin were measured and shown in Figure 1. For Somofilcon-A lenses, the COF in PBS was the most stable during the period of fifteen cycles (Figure 1a). The COF was 0.009 and 0.007 during the first cycle when Somofilcon-A lenses were sliding against the glass in 0.2 and 50 mg/mL albumin, respectively, but it dropped to 0.002 and 0.003 starting at cycle two (Figure 1a). A similar phenomenon was observed for Senofilcon-A lenses. The COFs were both 0.004 when Senofilcon-A lenses were sliding in 0.2 and 50 mg/mL albumin but decreased to 0.003 in cycle 2 and 3, respectively (Figure 1b). The COF of Etafilcon-A lenses was 0.007 during the first cycle but dropped below 0.003 afterwards when sliding in 0.2 mg/mL albumin and had stable COFs when sliding in PBS or 50 mg/mL albumin (Figure 1d). In contrast, the COF increased in cycle 3 when Polymacon lenses were sliding in PBS or 50 mg/mL albumin solution. The COF of Polymacon lenses in 0.2 mg/mL albumin was relatively stable (Figure 1c).

### 3.2. Coefficients of Friction of Contact Lenses after Being Immersed in a High Concentration of Albumin

**Figure 2:**
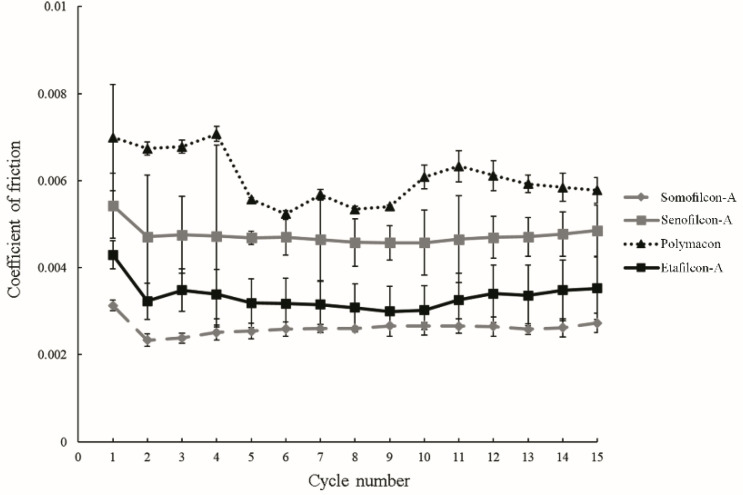
The coefficients of friction of Somofilcon-A (gray dashed line), Senofilcon-A (gray line), Polymacon (black dotted line), and Etafilcon-A (black line) in 50 mg/mL albumin solution after contact lenses are immersed in 50 mg/mL albumin for 15 min. Error bars represented standard deviation, and four experiments were used for calculating error bars.

All contact lenses were immersed in 50 mg/mL albumin for fifteen minutes and then were slid against the glass in 50 mg/mL albumin for fifteen cycles. The results showed the decrease of COF from cycle 1 to cycle 2 for all the lens materials (Figure 2). The COF of Polymacon lenses was the highest (all above 0.005), while the COF of Somofilcon-A lenses was the lowest (all below 0.003).

### 3.3. Albumin Deposition on the Contact Lenses

**Figure 3:**
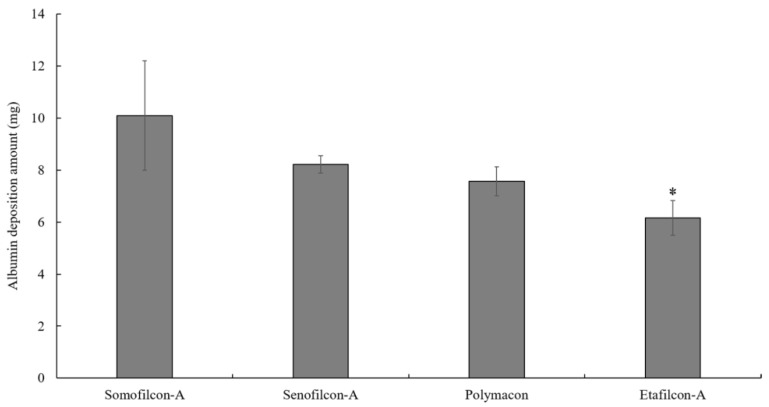
Deposited albumin concentrations are measured after Somofilcon-A, Senofilcon-A, Polymacon, and Etafilcon-A lenses are immersed in 50 mg/mL albumin solution for 15 min. * *p* < 0.05 when comparing albumin deposition amount on Senofilcon-A versus on Etafilcon-A lenses. Error bars represented standard deviation and were obtained from three experiments.

To investigate the amount of albumin deposition on contact lenses prior to the friction testing, contact lenses were immersed in 50 mg/mL albumin for 15 min. Somofilcon-A displayed the highest albumin deposition amount, while Etafilcon-A showed the lowest deposition amount, but there was no statistical difference (Figure 3).

### 3.4. Surface Roughness

**Figure 4:**
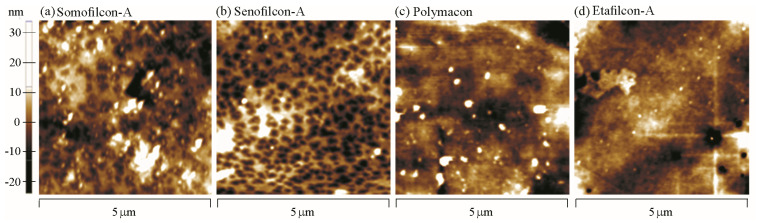
Surface roughness analysis for Somofilcon-A (**a**), Senofilcon-A (**b**), Polymacon (**c**), and Etafilcon-A (**d**) lenses.

The surface of Etafilcon-A was the smoothest, and the average roughness (Ra) was 0.564 μm (Figure 4d). The Somofilcon-A lens had the roughest surface with an Ra of 4.864 μm (Figure 4a). The Ra values for Senofilcon-A and Polymacon lenses were 3.254 μm and 2.478 μm, respectively (Figure 4b–c).

## 4. Discussion

We analyzed the role of albumin deposition on the coefficients of friction (COF) of four contact lens materials in this study. When contact lenses were sliding in albumin solution immediately after being taken out of the packaging, it mimicked the conditions of when contact lenses are initially put into the eyes. The result showed that the COF was relatively high during the first sliding minute for Somofilcon-A and Etafilcon-A lenses in 0.2 mg/mL albumin, which is similar to the concentration of albumin after wearing contact lenses [17]. Since the in vitro COF of contact lenses could correspond to in vivo comfort degree [10,11], it may suggest that wearers would feel some degree of discomfort when they first wear Somofilcon-A or Etafilcon-A lenses. However, the COF was subsequently reduced for both Somofilcon-A and Etafilcon-A lenses, suggesting that wearers would not feel discomfort once their eyes adapted to the contact lenses. In summary, the COFs of all contact lenses in 0.2 mg/mL albumin solution were lower than in PBS. When contact lenses were sliding in 50 mg/mL albumin, the COF of Somofilcon-A was even lower than when sliding in 0.2 mg/mL albumin; thus, we speculate that albumin might act as a lubricant for these four contact lens materials.

The protein conformational change, but not the amount of protein deposition on the contact lens, has been shown to correspond to comfort [18,19]. Indeed, the albumin deposition analysis showed that the amount of albumin deposited on Somofilcon-A was the highest when lenses were immersed in 50 mg/mL albumin for 15 min. However, the COF of Somofilcon-A lenses was lowest when sliding in 50 mg/mL albumin solution after being immersed in 50 mg/mL albumin for 15 min. The COF of Polymacon lenses was the highest in albumin solution, but the amount of albumin deposition was the second lowest. Although the measurement of albumin deposition on the lens in this study was not the most accurate method of protein adsorption, the results demonstrated that the amount of albumin deposition did not appear to correspond to the COF. Therefore, the mechanism of how albumin undergoes a conformational change after deposition during friction needs to be considered.

**Figure 5:**
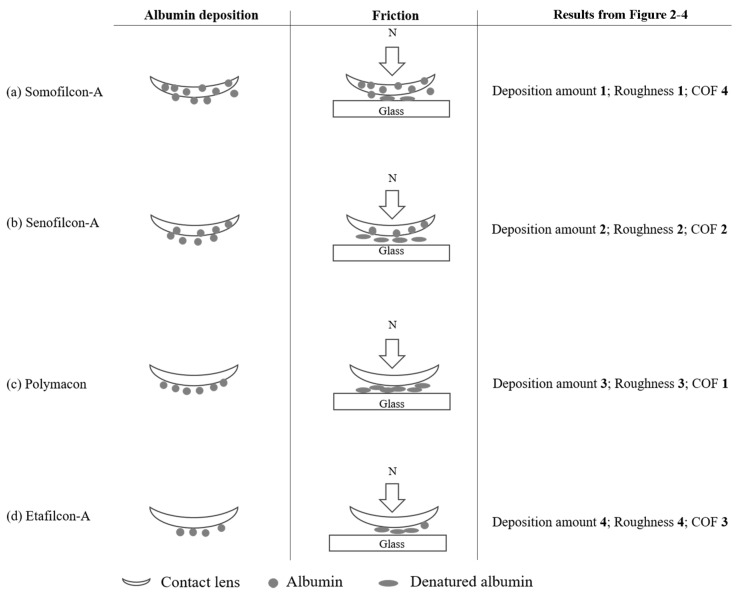
The potential model of how different materials of contact lenses display distinct behavior of albumin deposition and tribological properties. (**a**) Majority of albumin may permeate into the matrix of Somofilcon-A lens, resulting in the highest albumin deposition amount but the lowest COF. (**b**) Although the rough surface of Senofilcon-A results in a large amount of adsorbed albumin, friction still causes albumin on the surface to undergo conformational change, leading to an increased COF. (**c**) Albumin is only adsorbed on the surface and may undergo the conformational change resulting in the highest COF of Polymacon lens even though the amount of albumin deposition is the second lowest. (**d**) A lower amount of albumin deposition on the surface of Etafilcon-A results in a lower COF. In the column of results from Figure 2, Figure 3 and Figure 4, the number represents ranking. For example, deposition amount 1 represents the highest amount of albumin deposition.

Proteins are first in solution and move toward the contact lens surface; then, the proteins are adsorbed to the surface followed by structural changes [20]. It has been shown that the contact lens material composition, pore size, water content, hydrophobicity, surface roughness, contact lens or protein charge, protein size, etc. all play a role in protein deposition [5]. Then, we proposed a model that may explain the results (Figure 5). Both Somofilcon-A and Senofilcon-A lenses are silicone hydrogel contact lenses, but Senofilcon-A lenses are coated with vinyl pyrrolidone (PVP) as a wetting agent to reduce hydrophobicity [21]. Albumin has been demonstrated to be denatured on hydrophobic surfaces more easily than on hydrophilic surfaces [22]. However, the COF of Somofilcon-A lenses was lower than that of Senofilcon-A lenses, suggesting that surface hydrophobicity might not be a factor that affects albumin conformational change. Other factors could be the water content and surface roughness of contact lenses. Higher water content leads to larger pore sizes, which may result in protein penetration into the matrix of contact lenses [23,24]. Water content is 56% in Somofilcon-A lenses and 38% in Senofilcon-A lenses [25]; thus, the pore sizes of Somofilcon-A should be larger than those of Senofilcon-A. In addition, the surface roughness results showed that the Ra value of Somofilcon-A was 4.864 μm, while the Ra value of Senofilcon-A was 3.254 μm, suggesting there might be more deposits forming on imperfections of the surface. Taken together, it is possible that albumin was more likely to penetrate into the matrix of Somofilcon-A and get deposited on the imperfections of the surface, resulting in less albumin on the surface against the glass. Therefore, less albumin might undergo conformational changes on the surface, leading to a smaller COF of Somofilcon-A lenses compared to Senofilcon-A lenses (Figure 5a,b). However, higher water contents display lower oxygen permeability for silicone hydrogel contact lenses [26]. Wearing contact lenses with high oxygen permeability can reduce contact lens-induced hypoxia, resulting in better ocular physiology; thus, wearers may choose Senofilcon-A even though Somofilcon-A lenses provide better lubrication than was shown here. Therefore, more clinical investigation will be required to understand whether the in vitro friction coefficient of contact lenses could be directly corresponded to the in vivo comfort.

Both Polymacon and Etafilcon-A are materials used in hydrogel contact lenses, but Polymacon is hydrophobic, while Etafilcon-A has hydrophilic properties [27,28]. Water contents of Etafilcon-A and Polymacon are 58% and 38.6%, respectively [29]. It is possible that the low water content of Polymacon lenses causes albumin to stay on the surface, and albumin may go through conformational changes because of the hydrophobic properties of the material (Figure 5c). It might be the reason why the COF of Polymacon was higher than Etafilcon-A. In addition, Etafilcon-A is negatively charged [28], whereas the pI pH of albumin is 5.16 [5]. Therefore, albumin is not easily attracted or bound to Etafilcon-A lenses. The higher water content and electrical repulsion might cause albumin to either penetrate into the matrix or repel away from the surface, resulting in less albumin on the surface of Etafilcon-A lenses to undergo conformational changes, resulting in a lower COF (Figure 5d).

Under control tear albumin concentrations (0.2 mg/mL), albumin was able to provide lubrication for Somofilcon-A, Senofilcon-A, Polymacon, and Etafilcon-A lenses. The result showed that albumin acted distinctly from lysozyme, as we previously demonstrated that lysozyme would increase the COF of some hydrogel contact lenses [15,16]. However, only one protein was investigated here. Whether the effect of albumin alone on the tribological properties of contact lenses is the same as the impact of a mixture of tear proteins and whether the in vitro friction test results demonstrated here could relate to the in vivo bio-tribological property between the contact lens and the eyelid needs further investigation. Albumin has been used as eye drops for treating severe dry eye [30,31]. Since many contact lens wearers feel eyes are dry after a long period of time, albumin might be considered as a lubricating additive in the artificial tears. The dosages of albumin in the artificial tears need to be investigated when albumin is used as a lubricant without affecting ocular physiology.

## 5. Conclusions

The current study showed that under control tear albumin concentration, albumin acted as a lubricant for both silicone hydrogel and hydrogel lenses investigated here. Many factors may affect the COFs of contact lenses, but the results demonstrated that no correspondence was observed between the amount of deposited albumin or between the surface roughness and the tribological properties of contact lenses. The results suggested that albumin might be applied as a lubricating additive in the artificial tears and can be used for contact lens wearers when eyes feel dry after wearing for a long period of time.

## Data Availability

The data presented in this study are available on request from the corresponding author.
